# Molecular interactions of the chaperone CcmS and carboxysome shell protein CcmK1 that mediate β-carboxysome assembly

**DOI:** 10.1093/plphys/kiae438

**Published:** 2024-08-22

**Authors:** Jin Cheng, Chun-Yang Li, Meng Meng, Jian-Xun Li, Shu-Jun Liu, Hai-Yan Cao, Ning Wang, Yu-Zhong Zhang, Lu-Ning Liu

**Affiliations:** MOE Key Laboratory of Evolution and Marine Biodiversity, Frontiers Science Center for Deep Ocean Multispheres and Earth System & College of Marine Life Sciences, Ocean University of China, Qingdao 266003, China; Joint Research Center for Marine Microbial Science and Technology, Shandong University and Ocean University of China, Qingdao 266071, China; MOE Key Laboratory of Evolution and Marine Biodiversity, Frontiers Science Center for Deep Ocean Multispheres and Earth System & College of Marine Life Sciences, Ocean University of China, Qingdao 266003, China; Joint Research Center for Marine Microbial Science and Technology, Shandong University and Ocean University of China, Qingdao 266071, China; MOE Key Laboratory of Evolution and Marine Biodiversity, Frontiers Science Center for Deep Ocean Multispheres and Earth System & College of Marine Life Sciences, Ocean University of China, Qingdao 266003, China; Joint Research Center for Marine Microbial Science and Technology, Shandong University and Ocean University of China, Qingdao 266071, China; Joint Research Center for Marine Microbial Science and Technology, Shandong University and Ocean University of China, Qingdao 266071, China; Marine Biotechnology Research Center, State Key Laboratory of Microbial Technology, Shandong University, Qingdao 266237, China; MOE Key Laboratory of Evolution and Marine Biodiversity, Frontiers Science Center for Deep Ocean Multispheres and Earth System & College of Marine Life Sciences, Ocean University of China, Qingdao 266003, China; Joint Research Center for Marine Microbial Science and Technology, Shandong University and Ocean University of China, Qingdao 266071, China; MOE Key Laboratory of Evolution and Marine Biodiversity, Frontiers Science Center for Deep Ocean Multispheres and Earth System & College of Marine Life Sciences, Ocean University of China, Qingdao 266003, China; Joint Research Center for Marine Microbial Science and Technology, Shandong University and Ocean University of China, Qingdao 266071, China; MOE Key Laboratory of Evolution and Marine Biodiversity, Frontiers Science Center for Deep Ocean Multispheres and Earth System & College of Marine Life Sciences, Ocean University of China, Qingdao 266003, China; Joint Research Center for Marine Microbial Science and Technology, Shandong University and Ocean University of China, Qingdao 266071, China; MOE Key Laboratory of Evolution and Marine Biodiversity, Frontiers Science Center for Deep Ocean Multispheres and Earth System & College of Marine Life Sciences, Ocean University of China, Qingdao 266003, China; Joint Research Center for Marine Microbial Science and Technology, Shandong University and Ocean University of China, Qingdao 266071, China; Marine Biotechnology Research Center, State Key Laboratory of Microbial Technology, Shandong University, Qingdao 266237, China; Laboratory for Marine Biology and Biotechnology, Qingdao Marine Science and Technology Center, Qingdao 266237, China; MOE Key Laboratory of Evolution and Marine Biodiversity, Frontiers Science Center for Deep Ocean Multispheres and Earth System & College of Marine Life Sciences, Ocean University of China, Qingdao 266003, China; Institute of Systems, Molecular and Integrative Biology, University of Liverpool, Liverpool L69 7ZB, UK

## Abstract

The carboxysome is a natural proteinaceous organelle for carbon fixation in cyanobacteria and chemoautotrophs. It comprises hundreds of protein homologs that self-assemble to form a polyhedral shell structure to sequester cargo enzymes, ribulose 1,5-bisphosphate carboxylase/oxygenase (Rubisco), and carbonic anhydrases. How these protein components assemble to construct a functional carboxysome is a central question in not only understanding carboxysome structure and function but also synthetic engineering of carboxysomes for biotechnological applications. Here, we determined the structure of the chaperone protein CcmS, which has recently been identified to be involved in β-carboxysome assembly, and its interactions with β-carboxysome proteins. The crystal structure at 1.99 Å resolution reveals CcmS from *Nostoc* sp. PCC 7120 forms a homodimer, and each CcmS monomer consists of five α-helices and four β-sheets. Biochemical assays indicate that CcmS specifically interacts with the C-terminal extension of the carboxysome shell protein CcmK1, but not the shell protein homolog CcmK2 or the carboxysome scaffolding protein CcmM. Moreover, we solved the structure of a stable complex of CcmS and the C-terminus of CcmK1 at 1.67 Å resolution and unveiled how the CcmS dimer interacts with the C-terminus of CcmK1. These findings allowed us to propose a model to illustrate CcmS-mediated β-carboxysome assembly by interacting with CcmK1 at the outer shell surface. Collectively, our study provides detailed insights into the accessory factors that drive and regulate carboxysome assembly, thereby improving our knowledge of carboxysome structure, function, and bioengineering.

## Teaser

Crystal structures and biochemical analysis of the chaperone CcmS and carboxysome shell proteins reveal how they interact for β-carboxysome assembly in cyanobacteria.

## Introduction

Carboxysomes are self-assembling proteinaceous organelles found in cyanobacteria and chemoautotrophic bacteria, making a substantial contribution to the global CO_2_ cycle and microbial ecology (Turmo et al. 2017; Kerfeld et al. 2018; Liu 2022). The carboxysome sequesters the CO_2_-fixing enzyme, ribulose-1,5-bisphosphate carboxylase/oxygenase (Rubisco), and carbonic anhydrase (CA), within a polyhedral protein shell tiled with multiple protein paralogs in the forms of hexamers and trimers that constitute the facet of the shell, as well as pentamers that occupy the shell vertices (Kerfeld et al. 2005; Iancu et al. 2007; Tanaka et al. 2008; Klein et al. 2009; Cai et al. 2013; Rae et al. 2013; Keeling et al. 2014; Larsson et al. 2017; Sutter et al. 2017). The protein shell has selective permeability, ensuring the passage of HCO_3_^−^ and ribulose 1,5-bisphosphate (RuBP) through the central pores of shell proteins, whilst precluding O_2_ influx and leakage of CO_2_ from the carboxysome to the cytoplasm (Dou et al. 2008; Mahinthichaichan et al. 2018; Faulkner et al. 2020). Within the carboxysome, HCO_3_^−^ is dehydrated to CO_2_ by CA, resulting in elevated CO_2_ levels around Rubisco to enhance Rubisco carboxylation activities whilst alleviating unproductive oxygenation reactions (Price et al. 2008; Long et al. 2021). The intrinsic assembly and encapsulation mechanisms enable the formation of the polyhedral architecture of carboxysomes in the bacterial cytoplasm, providing the structural foundation for CO_2_ fixation as part of bacterial CO_2_-concentrating mechanism (Rae et al. 2013, 2017; Hennacy and Jonikas 2020). Moreover, the self-assembly discipline, structural modularity, and shell permeability make the carboxysomes an exceptional model system in synthetic biology for engineering catalytic nanoreactors and scaffolding nanomaterials, with the intent of enhancing cell metabolism and underpinning molecule delivery for various applications in biotechnology (Flamholz et al. 2020; Li et al. 2020; Chen et al. 2022; Jiang et al. 2023; Chen et al. 2023a, 2023b; Li et al. 2024).

According to the form of encapsulated Rubisco and protein composition, carboxysomes in different organisms can be divided into two types: α-carboxysomes and β-carboxysomes (Tabita, 1999; Badger et al. 2002; Badger and Bek 2008). α-Carboxysomes contain Form 1A Rubisco and their carboxysome-related genes are typically encoded in a single operon, whereas β-carboxysomes contain Form 1B Rubisco and tend to encode their carboxysome protein in multiple gene clusters (Tanaka et al. 2009). The β-carboxysome, in particular that in the model cyanobacterium *Synechococcus elongatus* PCC7942 (*Syn*7942), has been chosen as an important model system for studying carboxysome formation and function. The shell proteins of the β-carboxysome are encoded by a series of homologous *ccm*-type genes (Price and Badger 1991; Sun et al. 2019). The carboxysome shell hexameric proteins CcmKs are the most abundant shell protein components of β-carboxysomes and have various paralogs. For example, the β-carboxysome operons of *Synechocystis* sp. PCC6803 (*Syn*6803) and *Nostoc* sp. strain PCC 7120 (*Nos*7120) contain four CcmK genes (*ccmK1-4*) (Sommer et al. 2017). Other carboxysome shell proteins involve CcmL, CcmO, and CcmP. Moreover, the carboxysome scaffolding proteins CcmM and CcmN play important roles in permitting the nucleation of Rubisco and shell-cargo association (Eisenhut et al. 2007; Long et al. 2007, 2010; Kinney et al. 2012; Gonzalez-Esquer et al. 2015; Ryan et al. 2019; Wang et al. 2019).

Understanding how these building protein components assemble to form a functional carboxysome is vital for studies on carboxysome structure, function, and bioengineering. Previous work has elucidated that the β-carboxysome assembly exploited an “Inside out” pathway, which is initiated by Rubisco condensation with the assistance of CcmM, followed by shell encapsulation (Cameron et al. 2013; Chen et al. 2013). Despite these findings, further evidence has highlighted the requirement of external factors in de novo carboxysome biogenesis (Huang et al. 2019, 2020). Recent studies have identified the chaperone protein CcmS, which can interact with CcmK1, facilitating β-carboxysome assembly in *Syn*6803 (Chen et al. 2023b). However, the precise details of the CcmS structure and its interaction with β-carboxysome proteins to mediate β-carboxysome assembly remain unknown.

In this study, we solved the crystal structure of CcmS from *Nos*7120, revealing that CcmS forms a homodimer. Moreover, we solved the crystal structure of the stable complex of CcmS and the C-terminus of CcmK1 at 1.67 Å resolution. Our biochemical and structural results provide molecular insights into the association of CcmS with CcmK1 at the outer surface of the β-carboxysome shell, which facilitates the integration of CcmK1 and interactions of CcmK proteins within the shell during β-carboxysome shell assembly.

## Results

### Crystal structure of the CcmS dimer

**Figure 1.**
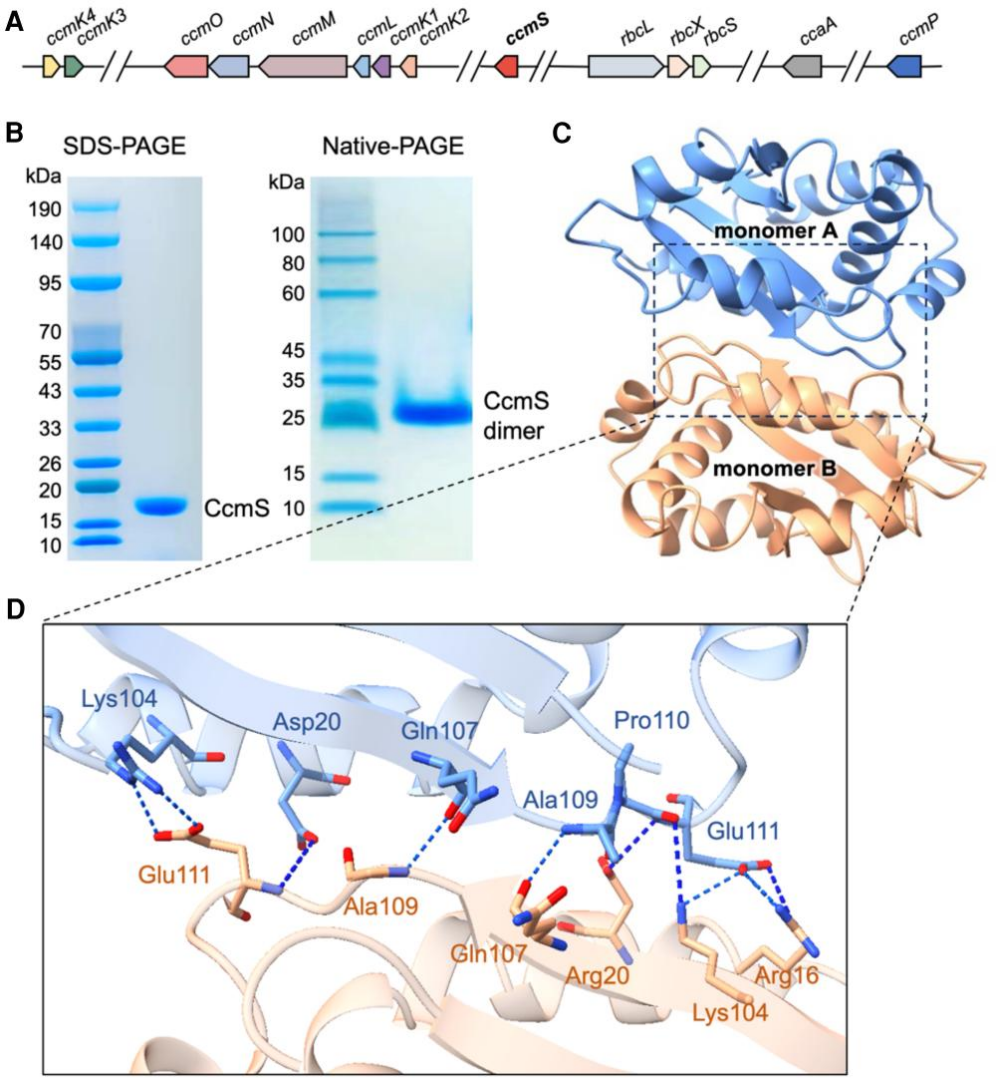
CcmS forms a dimer. **A)** The genomic arrangement of carboxysome-associated proteins in *Nostoc* sp. PCC 7120 (*Nos*7120). **B)** Purification of CcmS. SDS-PAGE and Native-PAGE analysis of the purified CcmS protein. **C)** Overall structure of the dimeric CcmS, in which monomer A (blue) and monomer B (orange) are labeled. **D)** Details of the dimer interface of CcmS. Overall structure of CcmS is shown as cartoon, whereas the interaction residues are shown as sticks. The possible interactions are indicated by dashed lines.

In the *Nos*7120 genome, the *ccmS* gene is located within the gene clusters expressing β-carboxysome proteins and carboxysome-related proteins (Fig. 1A). *ccmS* in *Nos*7120 encodes a protein of 142 amino acids. As determined by SDS-PAGE and Native-PAGE, CcmS functions as a dimer in solution, with the molecular mass of ∼16 kDa for each CcmS monomer (Fig. 1B). We solved the 1.99 Å crystal structure of the CcmS dimer in space group *P*2_1_2_1_2_1_ by molecular replacement (Supplementary Table S1 and Fig. S1). Two molecules of CcmS form a homodimer in the asymmetric unit, and each CcmS monomer consists of five α-helices and four β-sheets (Fig. 1C). The two CcmS molecules are rotated symmetrically, with an interaction interface of ∼900 Å^2^, primarily mediated by hydrogen bonds and salt bridges. Key interactions at the CcmS dimer interface are formed by Arg16-Glu111, Ala109-Gln107, Pro110-Lys104, Asp20-Glu111, and Lys104-Glu111 residues (Fig. 1D). These residues are highly conserved, suggesting that the dimerization of CcmS is ubiquitous among various β-cyanobacterial species (Supplementary Fig. S2).

### CcmS interacts with CcmK1 but does not interact with CcmK2 or CcmM

**Figure 2.**
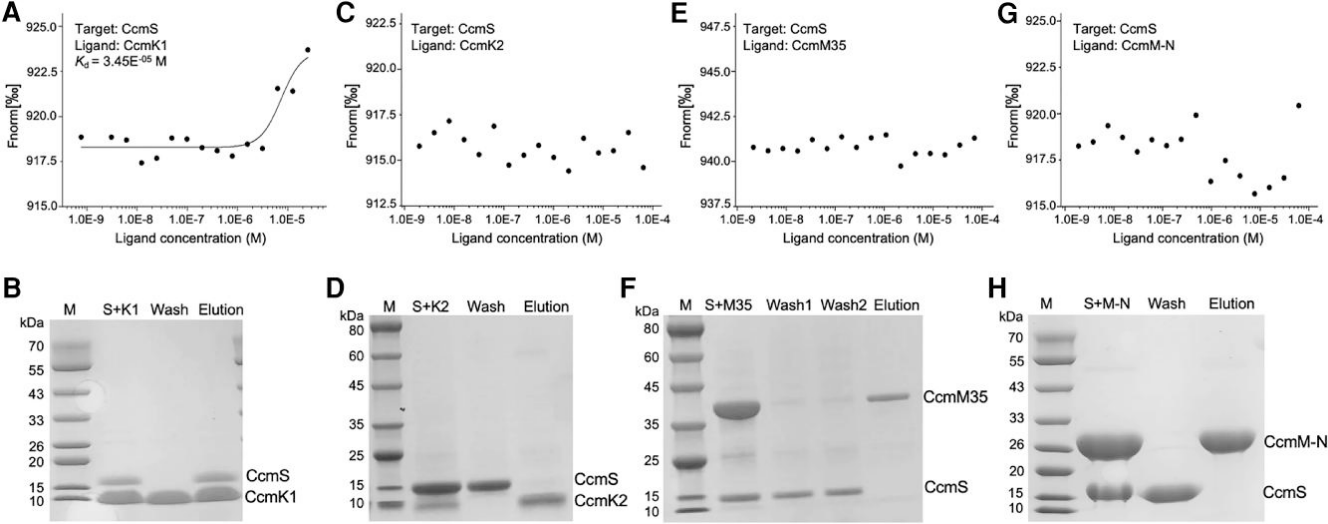
CcmS interacts with CcmK1 and CcmS does not interact with CcmK2, CcmM35, CcmM-N. **A)** MST analysis of CcmS and CcmK1. CcmS served as the target protein and CcmK1 was the ligand, with the concentrations of 0.25 and 25 *μ*m, respectively. The signal-to-noise ratio of MST analysis was 19.63 (>5). **B)** SDS-PAGE results of pull-down of CcmS and CcmK1. His-tagged CcmK1 was incubated with Strep-tagged CcmS. Protein mixtures were bound to the Strep-tag XT, then were washed and eluted for SDS-PAGE. **C)** MST analysis of CcmS and CcmK2. CcmS served as target protein and CcmK2 is the ligand, with the concentrations of 0.25 and 65 *μ*m, respectively. **D)** SDS-PAGE results of pull-down of CcmS and CcmK2. His-tagged CcmS was incubated with Strep-tagged CcmK2. **E)** MST analysis of CcmS and CcmM35. CcmS served as target protein and CcmM35 was the ligand, with the concentrations of 0.25 and 65 *μ*m, respectively. **F)** SDS-PAGE results of pull-down of CcmS and CcmM35. His-tagged CcmS was incubated with Strep-tagged CcmM35. **G)** MST analysis of CcmS and CcmM-N. CcmS served as target protein and CcmM-N was the ligand, with the concentrations of 0.25 and 62 *μ*m, respectively. **H)** SDS-PAGE results of pull-down of CcmS and CcmM-N. His-tagged CcmS was incubated with Strep-tagged CcmM-N.

To understand how CcmS participates in the assembly of β-carboxysomes, we examined the interactions between CcmS and β-carboxysome components, including the shell proteins CcmK1 and CcmK2 and the scaffolding protein CcmM, using microscale thermophoresis (MST) assays, pull-down assays, and gel filtration chromatography (Fig. 2; Supplementary Fig. S3). MST assays showed concentration-dependent changes in fluorescence values between CcmS and CcmK1 hexamers, indicating that CcmS dimers could interact with CcmK1 hexamers, with the dissociation constant (*K*_d_) of 34.5 *μ*m (Fig. 2A; Supplementary Figs. S4 and S5). This was further confirmed by CcmS–CcmK1 pull-down assays (Fig. 2B) and gel filtration chromatography (Supplementary Fig. S3). In contrast, MST assays indicated that CcmS did not form detectable interactions with CcmK2 (Fig. 2C). Supporting this result are the findings that CcmS dimers were washed off prior to the elution of CcmK2 in pull-down experiments (Fig. 2D) and gel filtration chromatography (Supplementary Fig. S3). Collectively, our results indicate that *Nos*7120 CcmS dimers can form strong interactions with CcmK1 hexamers, but not with CcmK2 hexamers. These results are consistent with the observations for *Syn*6803 (Chen et al. 2023b).

CcmS was also suggested to have a weak interaction with the scaffolding protein CcmM in *Syn*6803 (Chen et al. 2023b). The full-length form of CcmM consists of an N-terminal γ-carbonic anhydrase-like (γCAL) domain and the C-terminal small subunit-like (SSUL) modules (Cot et al. 2008; Peña et al. 2010). The shorter form of CcmM consists of only SSUL modules (Zang et al. 2021). In most β-carboxysomes, the γCAL domain is presumed to be responsible for recruiting the carboxysomal CA (CcaA) (Long et al. 2007; Cot et al. 2008), and forms interactions with the N-terminus of CcmN, which are crucial for the shell-cargo association (Kinney et al. 2012; Sun et al. 2021). The SSUL modules of CcmM mediate the formation of the Rubisco matrix by binding to the equatorial regions of Rubisco between the Ribulose bisphosphate carboxylase large subunit (RbcL) dimers (Ryan et al. 2019; Wang et al. 2019). In some cyanobacterial species, such as *Nos*7120, the β-carboxysomes lack CcaA homologs; instead, the γCAL domain of CcmM acts as a functional CA and does not play a role in recruiting CcaA (Peña et al. 2010; de Araujo et al. 2014). To determine the binding of CcmS with CcmM in *Nos*7120 in detail, we expressed the short-form CcmM (CcmM35, 35 kDa), which is the C-terminal region of CcmM, and the N-terminal region of CcmM (CcmM-N, 225 amino acid residues) of *Nos*7120, individually, and examined their interactions with CcmS. Our MST, pull-down, and chromatography analysis showed explicitly that there were no detectable interactions between CcmS and both the C-terminal and N-terminal regions of CcmM in *Nos*7120.

### CcmS interacts with the c-terminal fragment of CcmK1

**Figure 3.**
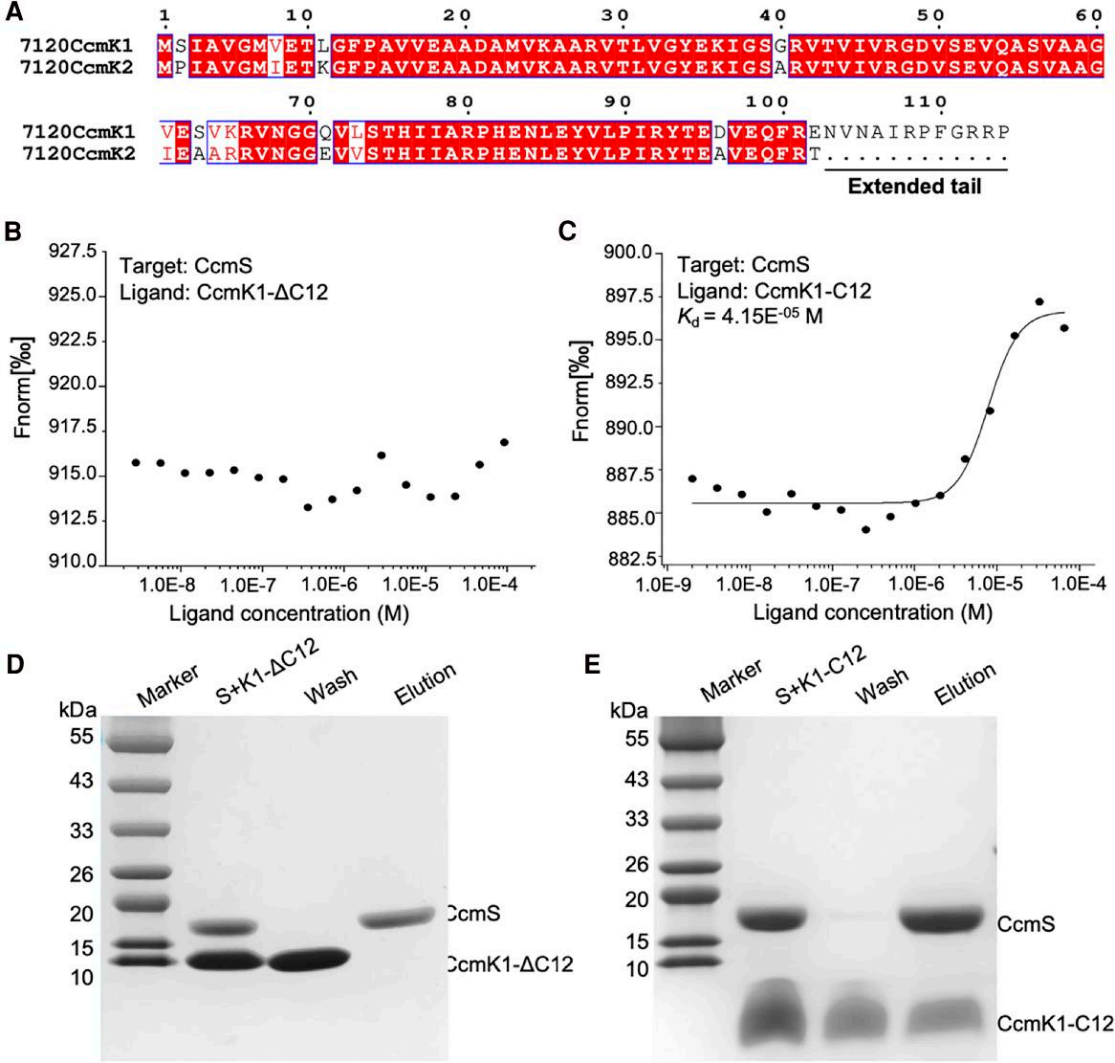
CcmS interacts specifically with the C-terminal extension of CcmK1. **A)** The amino sequence alignment between CcmK1 and CcmK2 from *Nos*7120. High similar residues (similarity higher than 70%) are framed, and residues with strict identity are highlighted in red. **B)** MST analysis of CcmS and CcmK1-ΔC12. CcmS served as target protein and CcmK1-ΔC12 was the ligand, with the concentrations of 0.25 and 92.5 *μ*m, respectively. **C)** MST analysis of CcmS and C-terminal peptides of CcmK1 (CcmK1-C12). CcmS served as target protein and CcmK1-C12 was the ligand, with the concentrations of 0.25 and 250 *μ*m, respectively. The signal-to-noise ratio was 17.26 (>5). **D)** Pull-down assays of CcmS and CcmK1-ΔC12 determined by SDS-PAGE. His-tagged CcmK1-ΔC12 was incubated with Strep-tagged CcmS. Protein mixtures were bound to Strep-tag XT, and then were washed and eluted for SDS-PAGE. **E)** Pull-down assays of CcmS and CcmK1-C12. CcmK1-C12 was incubated with Strep-tagged CcmS.

*Nos*7120 contains four CcmK paralogs, termed CcmK1-4. The *ccmK1* and *ccmK2* genes are located at the main *ccm* operon close to *ccmS* (Fig. 1A). The protein sequence identity between CcmK1 and CcmK2 is 89.11%, and the most notable difference is that CcmK1 has an extended C-terminal tail of 12 amino acids (Fig. 3A). Given the strong interaction between CcmK1 and CcmS and the lack of interaction between CcmK2 and CcmS (Fig. 2, A to D), we speculate that the C-terminal fragment of CcmK1 is responsible for its interaction with CcmS.

The C-terminus of CcmK1 is structurally disordered and exhibits the greatest structural changes among CcmK paralogs (Kerfeld et al. 2005; Tanaka et al. 2008; Tanaka et al. 2009). To verify the specific interaction between the CcmK1 C-terminus and CcmS, we generated a C-terminus-truncated CcmK1 (CcmK1-ΔC12), in which the C-terminal 12 amino acids were deleted. Gel filtration chromatography results indicated that CcmK1-ΔC12 formed hexamers, suggesting that the truncation of the CcmK1 C-terminus did not affect the assembly of the CcmK1 hexamer (Supplementary Figs. S4 and S5). As expected, interactions between CcmS and CcmK1-ΔC12 could not be detected (Fig. 3, B and D). Furthermore, we synthesized the 12-amino acid C-terminal peptide of CcmK1 (CcmK1-C12). Both MST analysis and pull-down results confirmed the strong interaction between CcmK1-C12 and CcmS (*K*_d_ = 41.5 *μ*m) (Fig. 3, C and E). Together, our results demonstrate that the C-terminus of CcmK1 drives its interaction with CcmS in *Nos*7120, consistent with previous studies in *Syn*6803 (Chen et al. 2023b).

### Structure of the CcmS/CcmK1 C-terminus complex

**Figure 4.**
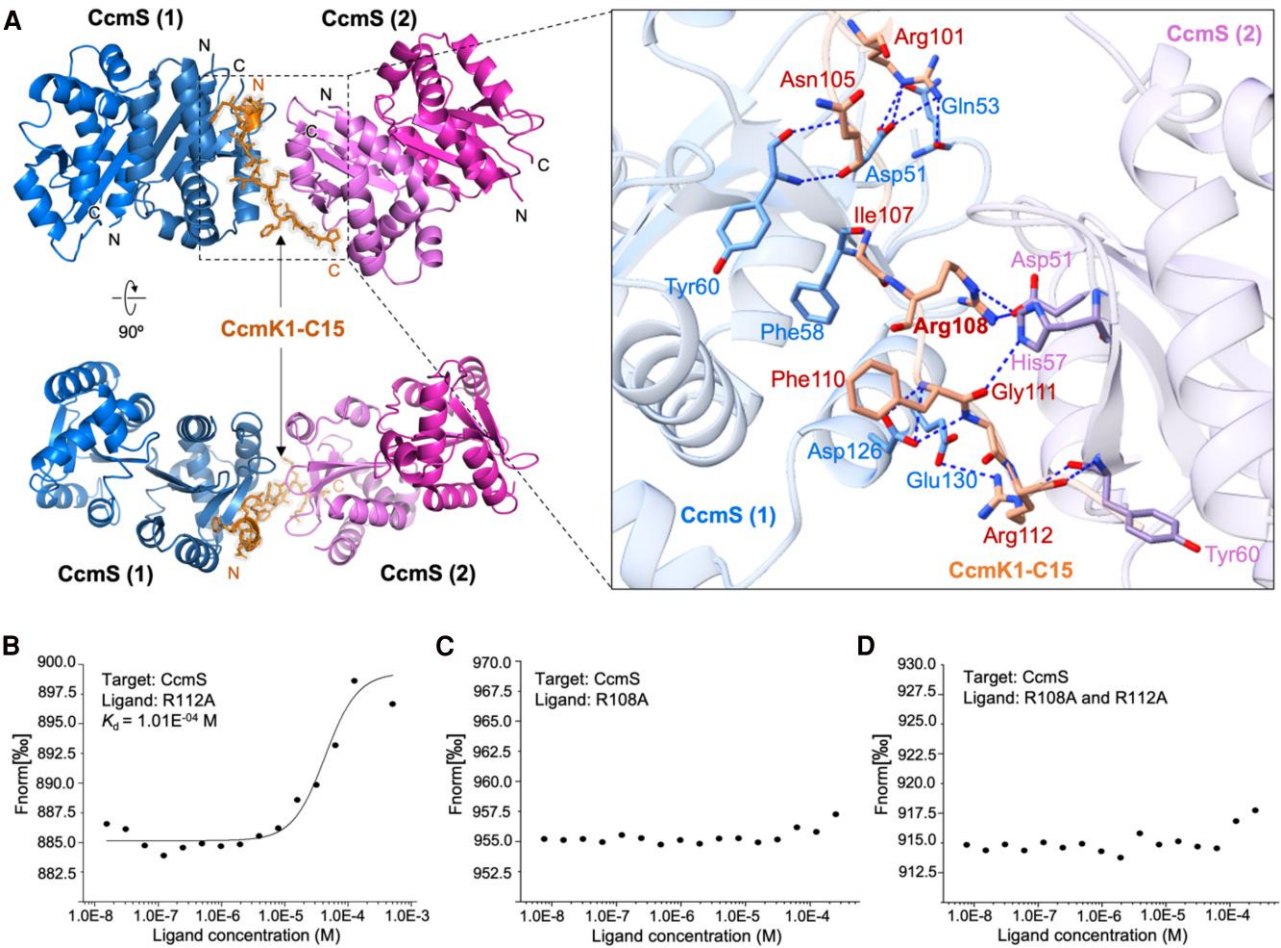
Structural analysis of the CcmS/CcmK1-C15 complex and key interacting residues. **A)** Left, crystal structure of two adjacent CcmS dimers shown in blue and purple ribbon representation, respectively (the two subunits of each dimer are also shown in different colors for clarity). The CcmK1-C15 between the two CcmS dimers is shown in orange ribbon representation with its electron density in gray at 1.5σ. Right, details of the interaction between the CcmS dimer and CcmK1-C15. The interaction residues are shown as sticks and the interactions are indicated by dashed lines. **B)** MST analysis of CcmS and R112A point mutations peptide. CcmS acted as target protein and the R112A peptide acted as ligand, with the concentrations of 0.25 and 500 *μ*m, respectively. **C)** MST analysis of CcmS and R108A peptide. CcmS acted as target protein and R108A peptide acted as ligand, with the concentrations of 0.25 and 500 *μ*m, respectively. **D)** MST analysis of CcmS and R108A and R112A double-mutant peptide. CcmS acted as target protein and the R108A/R112A double-mutant peptide acted as ligand, with the concentrations of 0.25 and 500 *μ*m, respectively.

To decipher how CcmS interacts with the C-terminal domain of CcmK1, we co-crystallized CcmS and the C-terminus of CcmK1 containing 15 amino acids (CcmK1-C15). After a period of crystal growth, we obtained high-quality crystals and solved the 1.67 Å crystal structure of the CcmS/CcmK1-C15 complex in space group *P*4_1_2_1_2 by molecular replacement (Supplementary Table S1 and Supplementary Fig. S1). In the refined structure, each asymmetric unit contains a CcmS dimer and a CcmK1-C15. CcmK1-C15 binds to one side of the CcmS dimer along the longitudinal direction through hydrogen bonds and salt bridges (Fig. 4A). The polar contacts between CcmK1-C15 and CcmS involve Arg101-Asp51, Arg101-Gln53, Ile107-Phe58, Asn105-Tyr60, Phe110-Asp126, Gly111-Asp126, and Arg112-Glu130 interactions. These findings highlight the strong interaction between the CcmS dimer and CcmS/CcmK1-C15, which are consistent with our biochemical results and previous findings (Chen et al. 2023b). Furthermore, these amino acid residues involved in the interactions between CcmS and CcmK1-C15 are conserved (Supplementary Figs. S2 and S6), implying a general interaction manner of CcmS and CcmK1 among different cyanobacterial species.

Intriguingly, superposition of the crystal structures of CcmS alone and the CcmS/CcmK1-C15 complex revealed that the binding with CcmK1-C15 resulted in conformational changes of CcmS. In particular, the α-helices in the W38-P89 region underwent a rotational shift, including the conformational change from a random curl to an α-helical structure (Video 1; Supplementary Fig. S7). Additionally, the β-sheets in the I98-I103 region near CcmK1-C15 became less ordered (Supplementary Fig. S7). These conformational alterations may be important for the binding and release of CcmS to and from CcmK1.

To verify the key residues involved in the CcmS–CcmK1 interactions, we further synthesized three CcmK1-C15 peptides with R108A and R112A point mutations. MST analysis showed that the R112A peptide still formed interactions with CcmS (Fig. 4B). In contrast, there was no detectable binding between CcmS and the R108A CcmK1-C15 peptide (Fig. 4C), or between CcmS and the R108A/R112A double mutant peptide (Fig. 4D). These results indicate that Arg108 of CcmK1 plays a pivotal role in mediating the binding between CcmS and CcmK1.

In the crystal structure of the CcmS/CcmK1-C15 complex, we found that one CcmK1-C15 peptide could also bind to the CcmS dimer of the neighboring asymmetric unit via Arg108-Asp51, Gly111-His57, and Arg112-Tyr60 interactions (Fig. 4A). These CcmS residues are highly conserved (Supplementary Fig. S2), suggesting that this binding may be universal in diverse cyanobacteria. CcmK1 from *Nos*7120 forms a hexamer (Supplementary Figs. S4 and S5), like CcmK1 from *Syn*6803 and other cyanobacterial species (Tanaka et al. 2008, 2009; Sutter et al. 2019), given their high sequence similarity (Supplementary Fig. S6). Our results showed that a single C-terminus of the CcmK1 subunit could bind to two CcmS dimers at two distinct interfaces, suggesting that one CcmK1 hexamer could potentially interact with multiple CcmS dimers. Gel filtration chromatography and Native-PAGE analysis revealed that the molecular mass of the CcmS/CcmK1 complex is about 133 to 158 kDa (Supplementary Figs. S3B and S5A), which suggests that the CcmS/CcmK1 complex likely consists of two CcmS dimers (∼32 kDa per dimer) and one CcmK 1 hexamer (∼73 kDa) (Fig. 1B; Supplementary Figs. S4 and S5). Additionally, CcmS dimers may also bind to other CcmK1 hexamers through their C-termini. These extensive interactions could aid in the association of multiple CcmK1 proteins and their incorporation into the shell during shell assembly.

## Discussion

CcmS has been recently reported to play a role in mediating the assembly of β-carboxysomes in *Syn*6803 (Chen et al. 2023b). Deletion of *ccmS* reduces the accumulation and assembly of CcmK1, leading to abnormal carboxysomes and slow growth of *Syn*6803. To uncover how CcmS interacts with CcmK1, in this work, we solved the crystal structure of CcmS and revealed that CcmS functions as a dimer of two CcmS monomers, with each monomer composed of five α-helices and four β-sheets (Fig. 1C). Given that the residues at the dimer interface are highly conserved, we speculate that CcmS are present as dimers among diverse β-cyanobacterial species. Furthermore, we show that the C-terminal extension of CcmK1 provides specific binding sites for CcmS dimers. Structural and biochemical analysis of the CcmS/CcmK1-C complex provides mechanistic insights into the precise interactions between CcmS and CcmK1, which are important for the assembly of the β-carboxysome shell.

The β-carboxysomes have several CcmK paralogs (CcmK1, CcmK2, CcmK3, CcmK4), and the exact composition of β-carboxysomes varies among cyanobacteria. While CcmK1 is likely one of the dominant shell proteins, it is not present in all β-carboxysomes, for example, *Syn*7942 β-carboxysomes. The co-existence of *ccmS* and *ccmK1* in cyanobacterial genomes suggests the close functional and evolutionary relationship between CcmS and CcmK1 (Chen et al. 2023b). CcmK1 possesses a unique 12-amino acid C-terminal extension (Kerfeld et al. 2005; Tanaka et al. 2008; Tanaka et al. 2009). As this C-terminal extension of CcmK1 is flexible and highly disordered, it has not been structurally determined in any previously reported CcmK1 structures (Tanaka et al. 2008; Tanaka et al. 2009; Sutter et al. 2019). Likewise, the function of the C-terminal extension of CcmK1 remains poorly understood, despite the fact that it has been found to alert the packing of CcmK layers and hence participate in protein–protein interactions (Tanaka et al. 2009). Our results and recent findings indicate that in both *Nos*7120 and *Syn*6803, CcmS can interact specially with the 12-amino acid CcmK1 C-terminus, and it does not interact with CcmK1 lacking the C-terminus or its homolog CcmK2 (Chen et al. 2023b). These findings further verify the functional and evolutionary links between CcmS and CcmK1. The amino acid residues involved in the interaction of CcmS with CcmK1-C15, including the key residue Arg108, are conserved, indicating that the specific binding between CcmS and CcmK1 is widespread across many β-cyanobacterial strains that contain CcmS and CcmK1. This may serve as an effective regulatory mechanism, facilitating the defined assembly of β-carboxysomes and fitness of bacteria in specific ecological niches. Similarly, multiple shell paralogs (CsoS1A/B/C and CsoS4A/B) largely exist in the α-carboxysome linkage, and CsoS1B has an extended C-terminus of 12 amino acids compared with CsoS1A and CsoS1C (Tsai et al. 2007; Sun et al. 2022). These findings indicate the structural variations and modularity of various carboxysomes in general. Likewise, the extended C-terminal tails of certain viral capsid proteins have been documented to engage with external proteins, contributing to the viral life cycle, including assembly and nuclear transport (Kim et al. 2023).

**Figure 5.**
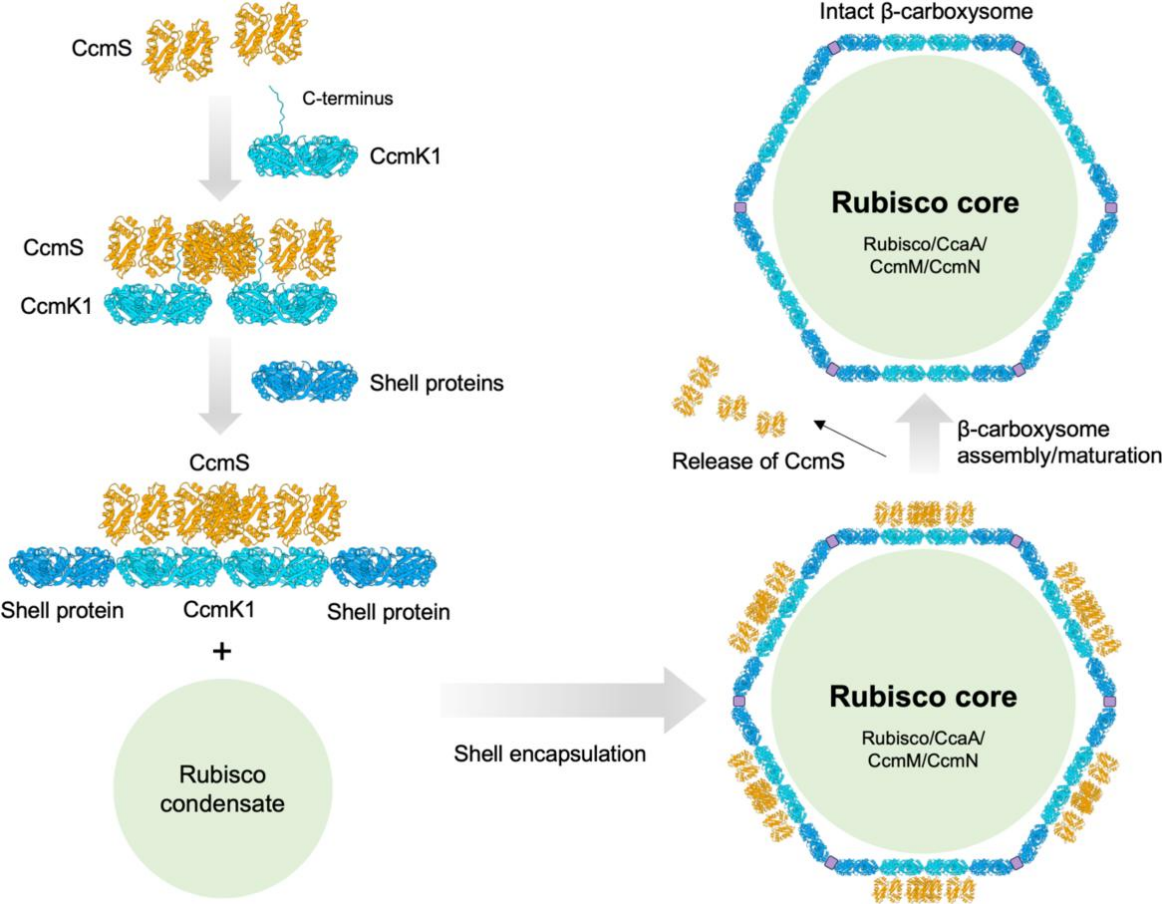
Conceptual model of CcmS-mediated β-carboxysome assembly. CcmS dimers specifically interact with the C-termini of CcmK1 hexamers on the concave surface of the shell. Each CcmK1 hexamer can bind to two CcmS dimers and CcmS dimers can also bind to neighboring CcmK1 hexamers, which eventually facilitate the assembly of several CcmK1 hexamers as small shell building blocks. This assembly may occur alongside the integration of CcmK2 and other shell proteins like CcmL, CcmO, and CcmP, ensuring the complete shell structure and enzyme encapsulation. Once the β-carboxysome is fully formed, CcmS dimers are released from the β-carboxysome shell into the cytoplasm.

Based on current knowledge, a model of CcmS-mediated β-carboxysome assembly is illustrated in Fig. 5. Following the in vivo folding and assembly of Rubisco, CcmM35 binds to the equatorial region of Rubisco L_8_S_8_ holoenzyme between the RbcL dimers. This results in the formation of Rubisco–CcmM complexes and Rubisco nucleation (Wang et al. 2019; Zang et al. 2021). Since β-carboxysome biogenesis undergoes the “Inside out” assembly pathway (Cameron et al. 2013), construction of the Rubisco matrix core and association with CcmN trigger the shell encapsulation. CcmS dimers interact specifically with CcmK1 hexamers at the C-termini at the concave surface of the shell facing toward the cytoplasm, and there are no interactions between CcmS and CcmK2 in the shell and between CcmS and CcmM in the carboxysome lumen. Each CcmK1 hexamer interacts with two CcmS dimers, forming a CcmS/CcmK1 complex, and the CcmS dimers within the complex could also bind to other CcmK1 hexamers. These interactions eventually facilitate the assembly of several CcmK1 hexamers to form small building blocks of shell facets. This process may occur simultaneously with the assembly of CcmK2 and other shell proteins CcmL, CcmO, and CcmP, to ensure the construction of an entire shell structure and enclosure of cargo enzymes. Moreover, previous studies on the model cyanobacteria *Syn*6803 and *Syn*7942 have suggested that different CcmK paralogs, such as CcmK1/K2 and CcmK3/K4, could form hetero-hexamers, which were proposed to play a role in fine-tuning the structural robustness and permeability of carboxysome shells (Garcia-Alles et al. 2019; Sommer et al. 2019). It is also possible that CcmS is involved in the formation of CcmK1/K2 or CcmK3/K4 heterohexamers that have specific extended C-terminal tails. After the formation of an intact β-carboxysome, CcmS dimers are released from the β-carboxysome shell into the cytoplasm, instead of becoming a permanent component of the β-carboxysome. The detailed mechanisms underlying CcmS-mediated β-carboxysome assembly and whether the conformational changes of CcmS drive the binding to or release from CcmK1 remain to be further elucidated. Moreover, since CcmS and CcmK1 are not present in all β-cyanobacteria, there must be alternative mechanisms for efficiently modulating the highly dynamic assembly of β-carboxysomes that do not contain CcmK1, which merits further investigation.

In summary, our findings provide structural insights into how auxiliary factors including CcmS mediate β-carboxysome assembly in many cyanobacteria. In translational terms, a comprehensive understanding of the mechanisms underlying carboxysome assembly and regulation offers important information to aid in the bioengineering of β-carboxysomes in heterologous hosts for various biotechnological applications, such as engineering functional β-carboxysomes into plants for improved photosynthesis and constructing carboxysome-based nanobioreactors into industrial bacterial species for enhanced catalytic performance and molecule delivery.

## Materials and methods

### Protein expression and purification

The *ccmS*, *ccmM35*, *ccmM*, *ccmK1*, and *ccmK2* genes of *Nostoc* sp. PCC 7120 (*Nos*7120) were synthesized by Shanghai Sangon Bioengineering Company and were inserted into pET-22b (+) with a His tag or Strep tag. Using the *ccmM* plasmid as a template, a pair of primers (*ccmM-N*-F/*ccmM-N*-R) containing restriction enzyme sites (*Nde* I and *Xho* I) and gene-specific sequences were used to amplify the *ccmM-N* fragment, and the *ccmM-N* gene fragment was ligated to the expression vector pET-22b (+) using a seamless cloning kit. Primers used in this study are listed in Supplementary Table S2.

The gene expression plasmid construction was carried out in *Escherichia coli* strain BL21(DE3) (Vazyme Biotech) at 37 °C in the lysogeny broth (LB) medium with 100 *μ*g mL^−1^ ampicillin. The *E. coli* strain BL21(DE3) constructs were cultured overnight at 37 °C in LB medium with 100 *μ*g mL^−1^ ampicillin to an OD_600_ of 0.8 to 1.0 and then induced at 25 °C for 12 to 16 h with 0.4 mm isopropyl β-D-1-thiogalactopyrano-side. The proteins were first purified with Ni^2+^-NTA resin (GE Healthcare, USA) or Strep-tag XT (IBA Lifescience, Germany) and then gel filtration chromatography was performed on a Superdex G75 column or a Superdex G200 column with FPLC (AKTA purifier 10, GE Healthcare, USA). Samples were incubated in advance to gel filtration chromatography.

### Sequence alignment

Protein sequences were downloaded from the UniProt and NCBI databases. The amino acid sequence alignments were aligned using CLUSTALW, and the results were modified using ESPript 3.0 (Robert and Gouet 2014).

### Strep-pull down assays

Purified CcmS-His proteins were mixed with CcmM35-Strep, CcmK2-Strep, or CcmM-N at a concentration ratio of 1:2 and were incubated overnight. The protein mixture was then loaded onto a Strep-tag XT column. After a 3-h incubation on the column, the protein mixture was washed through the column and the His-tagged proteins were eluted with wash buffer (10 mm Tris–HCl, pH8.0, 150 mm NaCl, 1 mm EDTA) and detected by Coomassie Brilliant Blue. The remaining proteins were eluted with elution buffer (10 mm Tris–HCl, pH8.0, 150 mm NaCl, 1 mm EDTA, 50 mm biotin). CcmS-Strep and CcmK1-His were mixed at a concentration ratio of 2:1, and other operations were the same as described above. All the protein samples were collected for SDS-PAGE analysis.

### Microscale thermophoresis

MST assays were performed as previously described (Jerabek-Willemsen et al. 2011). Dyes were incubated with target proteins at a final concentration of 0.25 *μ*m for 30 min at room temperature to label His-tagged target proteins. After mixing target proteins and 2-fold increased concentrations of nonlabeled legend proteins, the samples were drawn into 16 glass capillaries and were measured using a Monolith NT.115 instrument (Nano Temper Technologies GmbH, Munich, Germany) at 25 °C with 60% excitation power and medium LED power. The dissociation constant (*K*_d_) was calculated and fitted using the Nano Temper Analysis software.

### Crystallization and structure determination

Purified CcmS proteins were concentrated to ∼5 mg mL^−1^. Diffraction-quality crystals of the CcmS protein were obtained using the sitting-drop vapor diffusion method, in 0.2 m magnesium formate and 20% w/v PEG 3350 at 18 °C. X-ray diffraction data were collected on the BL19U1 beamline of the National Facility for Protein Sciences Shanghai (NFPS) and Shanghai Synchrotron Radiation Facility (SSRF) (Winn et al. 2011). CcmS crystals belong to space group *P*2_1_2_1_2_1_ with unit cell dimensions of *a* = 40.54, *b* = 74.63, *c* = 93.57, *α* = *β* = *γ* = 90° (Supplementary Table S1). To obtain the crystals of the complex of CcmS and CcmK1-C15, purified CcmS proteins were mixed with CcmK1-C15 at a molar ratio of 1:10, and the mixture was incubated overnight at 4 °C. Crystals of the CcmS/CcmK1-C15 complexes were obtained using the sitting-drop vapor diffusion method in 0.2 m lithium sulfate, 0.1 m Tris pH8.5, 1.26 m ammonium sulfate at 18 °C. X-ray diffraction data were collected on the BL18U1 beamline of NFPS and SSRF (Winn et al. 2011). Crystals of the CcmS/CcmK1-C15 complexes belong to space group *P*4_1_2_1_2 with unit cell dimensions of *a* = 67.49, *b* = 67.49, *c* = 118.11, *α* = *β* = *γ* =90°. Structures were solved by molecular replacement with CCP4 (McCoy et al. 2007) using a structure predicted by AlphaFold2 (Jumper et al. 2021) as a search model. Structure refinement was conducted using PHENIX (Afonine et al. 2012) and real space refinement was performed using Coot (Emsley and Cowtan 2004). Images of protein structures were created using ChimeraX and PyMOL. A list of the parameters for data collection, processing, structure determination and refinement is provided in Supplementary Table S1.

### Accession numbers

Sequence data from this article can be found in the GenBank/EMBL data libraries under accession numbers BAB73249.1 (CcmS), BAB72824.1 (CcmK1), BAB72825.1 (CcmK2), and BAB72822.1 (CcmM).

## Supplementary data

The following materials are available in the online version of this article.

**Supplementary Figure S1.** Crystallographic packing of CcmS dimers and CcmS/CcmK1-C15 complexes in the crystalline lattices.

**Supplementary Figure S2.** Sequence alignment analysis of CcmS from different strains.

**Supplementary Figure S3.** Gel filtration chromatography profiles of CcmK1, CcmK2, CcmM35, CcmK1-ΔC12 with CcmS.

**Supplementary Figure S4.** Purification and characterization of CcmK1.

**Supplementary Figure S5.** Interactions between CcmS, CcmK1, and CcmK1-ΔC12.

**Supplementary Figure S6.** Sequence alignment analysis of CcmS and CcmK1 from *Nos*7120 and *Syn*6803, as well as CcmK1 from diverse cyanobacterial species.

**Supplementary Figure S7.** Comparison of the crystal structures of CcmS and the CcmS/CcmK1-C15 complex indicates the conformational changes of the CcmS dimer resulted from the binding of CcmK1-C15.

**Supplementary Table S1.** Crystal parameters, data collection, and structure refinement.

**Supplementary Table S2.** Primers used in this study.

## Supplementary Material

kiae438_Supplementary_Data

## Data Availability

The crystal structures of CcmS and CcmS in complex with CcmK1-C15 have been deposited in PDB under the accession codes of 8ZLH and 8ZLZ, respectively.

## Dive Curated Terms

The following phenotypic, genotypic, and functional terms are of significance to the work described in this paper:

protein CHEBI: CHEBI:36080

x-ray Gramene: EO:0007628
x-ray Araport: EO:0007628
