# Supplementary material for: Molecular interactions of the chaperone CcmS and carboxysome shell protein CcmK1 that mediate β-carboxysome assembly
Source: Plant Physiol. 2024 Aug 22;196(3):1778–87. doi: 10.1093/plphys/kiae438 (PMC11635287; doi:10.1093/plphys/kiae438)
Supplement: kiae438_Supplementary_Data [file kiae438_Supplementary_Data.zip › Supplemental Information_R2.pdf]

Supplementary Information

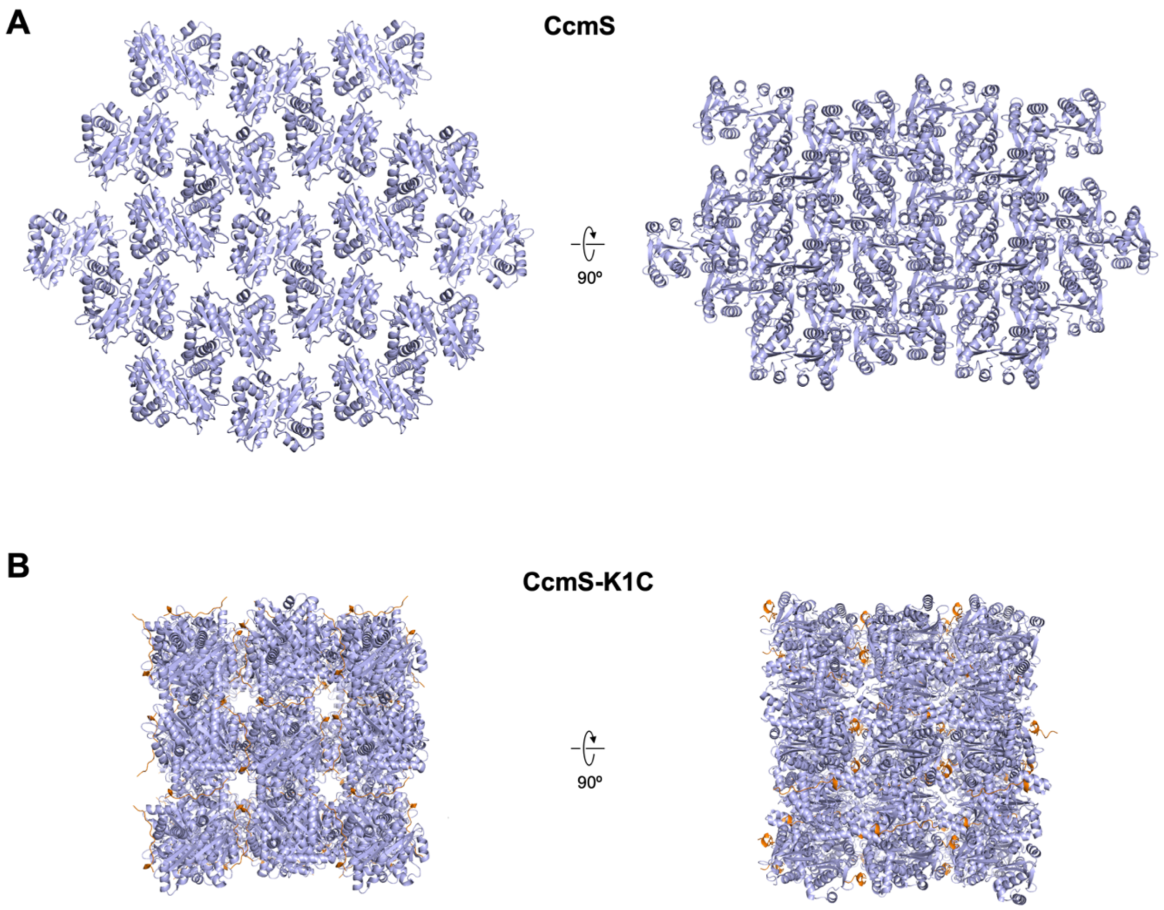

Supplementary Figure S1. Crystallographic packing of CcmS dimers and CcmS/CcmK1-C15 complexes in the crystalline lattices. A, Crystallographic packing of CcmS dimers in the crystalline lattices. CcmS crystals belong to space group $P2_12_12_1$ with unit cell dimensions of a=40.54, b=74.63, c=93.57, α=β=γ=90°. **B,** Crystallographic packing of CcmS/CcmK1-C15 complexes in the crystalline lattices. Crystals of the CcmS/CcmK1-C15 complexes belong to space group $P4_12_12$ with unit cell dimensions of a=67.49, b=67.49, c=118.11, α=β=γ=90° (see also Supplemental Table 1). CcmS dimers are displayed in light blue, and CcmK1-C15 peptides are displayed in orange. The figures were generated using PyMOL.

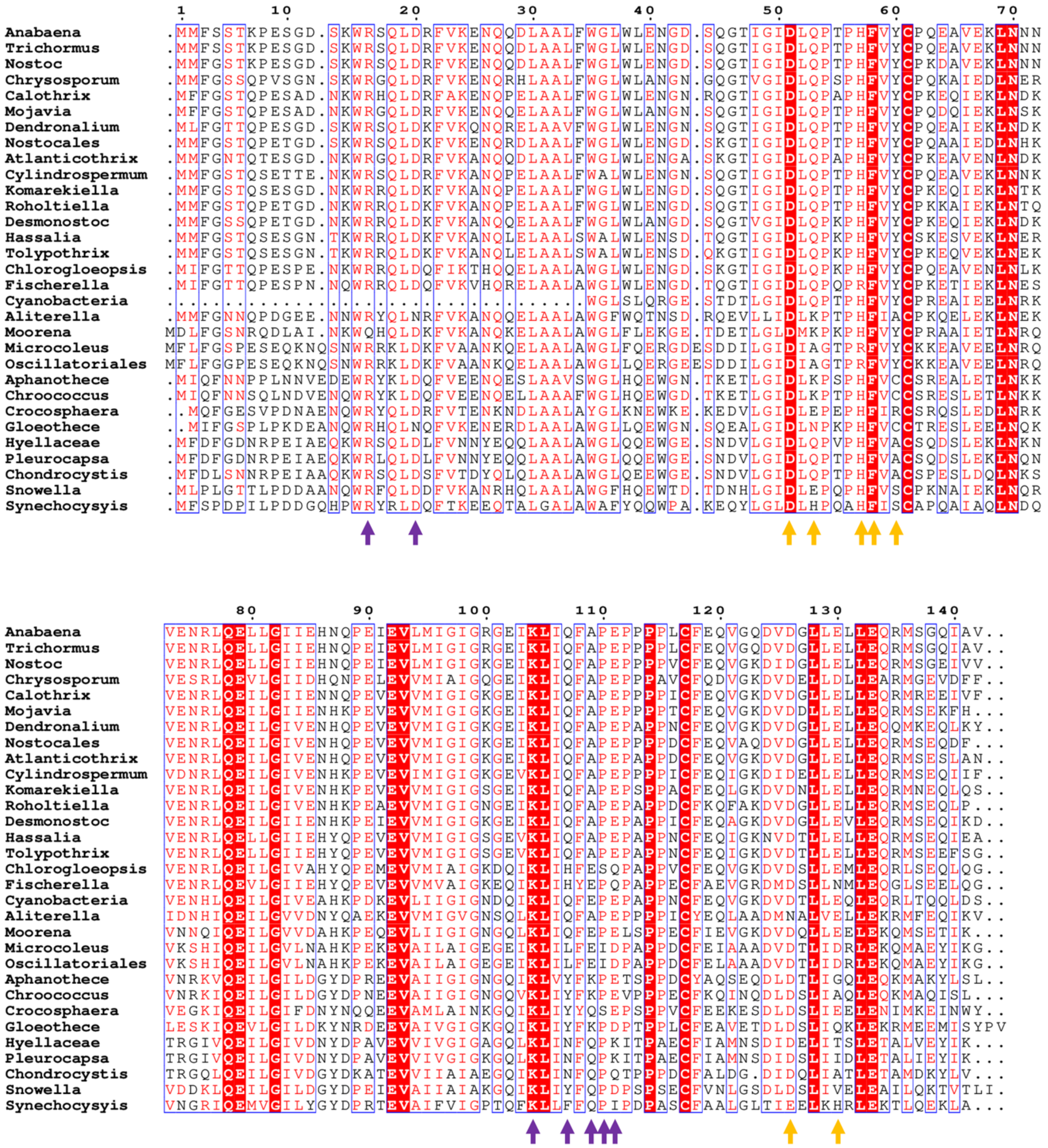

Supplementary Figure S2. Sequence alignment analysis of CcmS from different strains. High similar residues (similarity higher than 70%) are colored in red and framed in blue, residues with strict identity are in white on a red background. The amino acid residues Arg16, Asp20, Lys104, Gln107, Ala109, Pro10, Glu111 involved in the interaction at the CcmS dimer interface were marked by purple arrows, and the amino acids Asp51, Gln53, Tyr60, His57, Phe58, Asp126 and Gln130 involved in the interaction of CcmS with CcmK1C were marked by orange arrows.

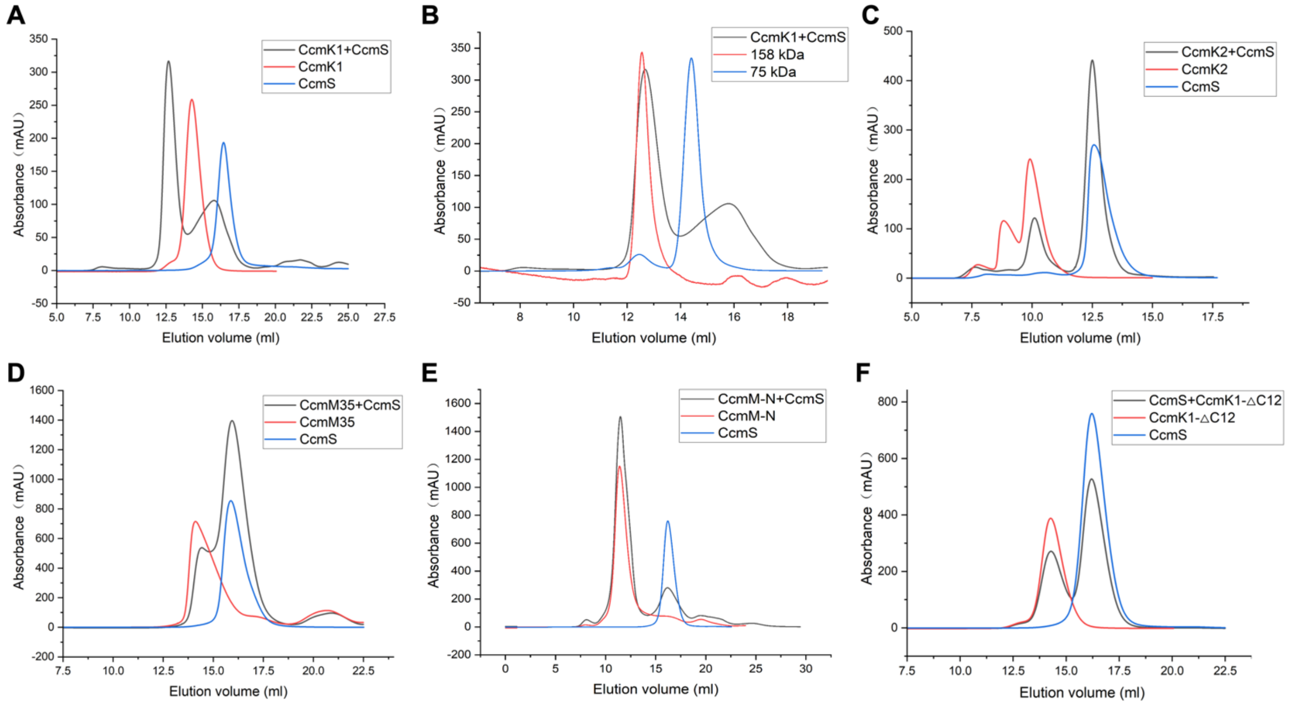

Supplementary Figure S3. Gel filtration chromatography profiles of CcmK1, CcmK2, CcmM35, CcmK1-ΔC12 with CcmS. A, Gel filtration chromatography profile of CcmK1 and CcmS. The purified proteins CcmK1 and CcmS were incubated overnight and were then subjected to gel filtration chromatography. **B,** Gel filtration chromatography profile of the CcmS/CcmK1 complex. Conalbumin (Mr = 75,000 Da, GE Healthcare) and Aldolase (Mr = 158,000 Da, GE Healthcare) were used as molecular mass markers. **C,** Gel filtration chromatography profile of CcmK2 and CcmS. **D,** Gel filtration chromatography profile of CcmM35 and CcmS. **E,** Gel filtration chromatography profile of CcmM-N and CcmS. **F,** Gel filtration chromatography profile of CcmK1-ΔC12 and CcmS.

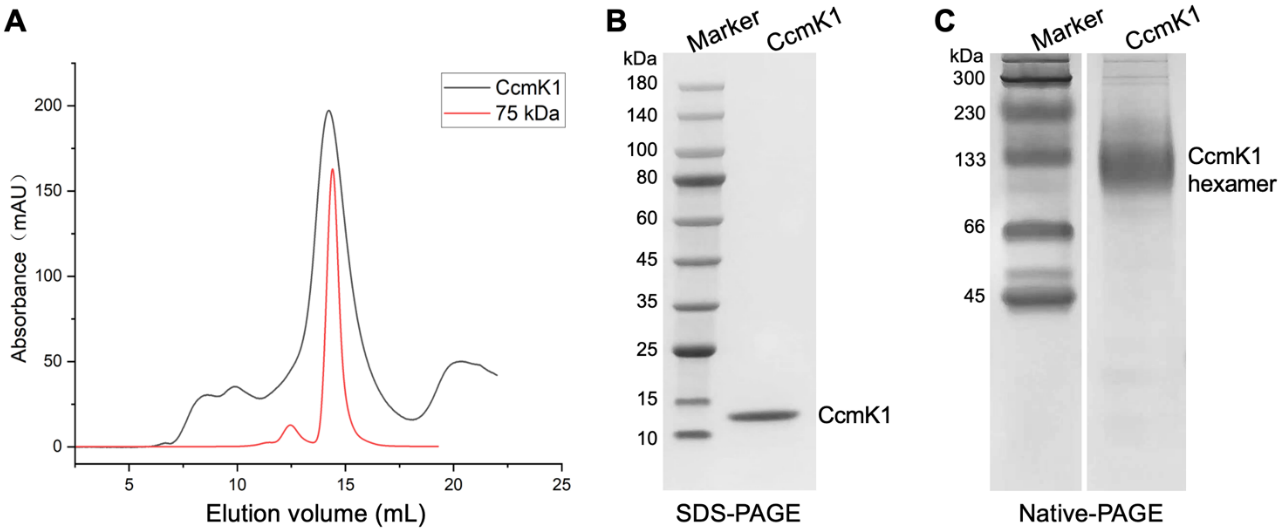

Supplementary Figure S4. **Purification and characterization of CcmK1. A**, Gel filtration chromatography of CcmK1, indicating that CcmK1 forms a hexamer. The protein of CcmK1 was subjected to Superdex G200 column. Conalbumin (Mr = 75,000 Da, GE Healthcare) was used as marker. The predicted molecular mass of CcmK1 is ~12,000 Da. **B**, SDS-PAGE analysis of CcmK1. **C**, Native-PAGE analysis of CcmK1 hexamer.

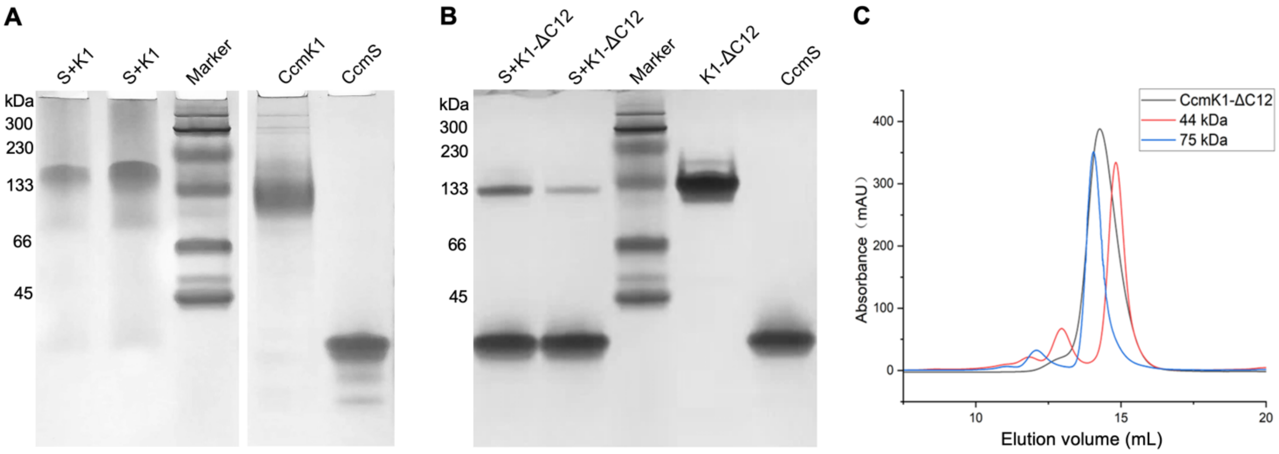

Supplementary Figure S5. Interactions between CcmS, CcmK1, and CcmK1-ΔC12. A, Native-PAGE of CcmS+CcmK1, CcmK1, and CcmS. CcmS and CcmK1 were incubated overnight before gel electrophoresis. **B,** Native-PAGE of CcmS+CcmK1-ΔC12, CcmK1-ΔC12, and CcmS. CcmS and CcmK1-ΔC12 were incubated overnight before gel electrophoresis. **C,** Gel filtration chromatography result of CcmK1-ΔC12. Conalbumin (Mr = 75,000 Da, GE Healthcare) and Ovalbumin (Mr = 44,000 Da, GE Healthcare) were used as molecular mass markers.

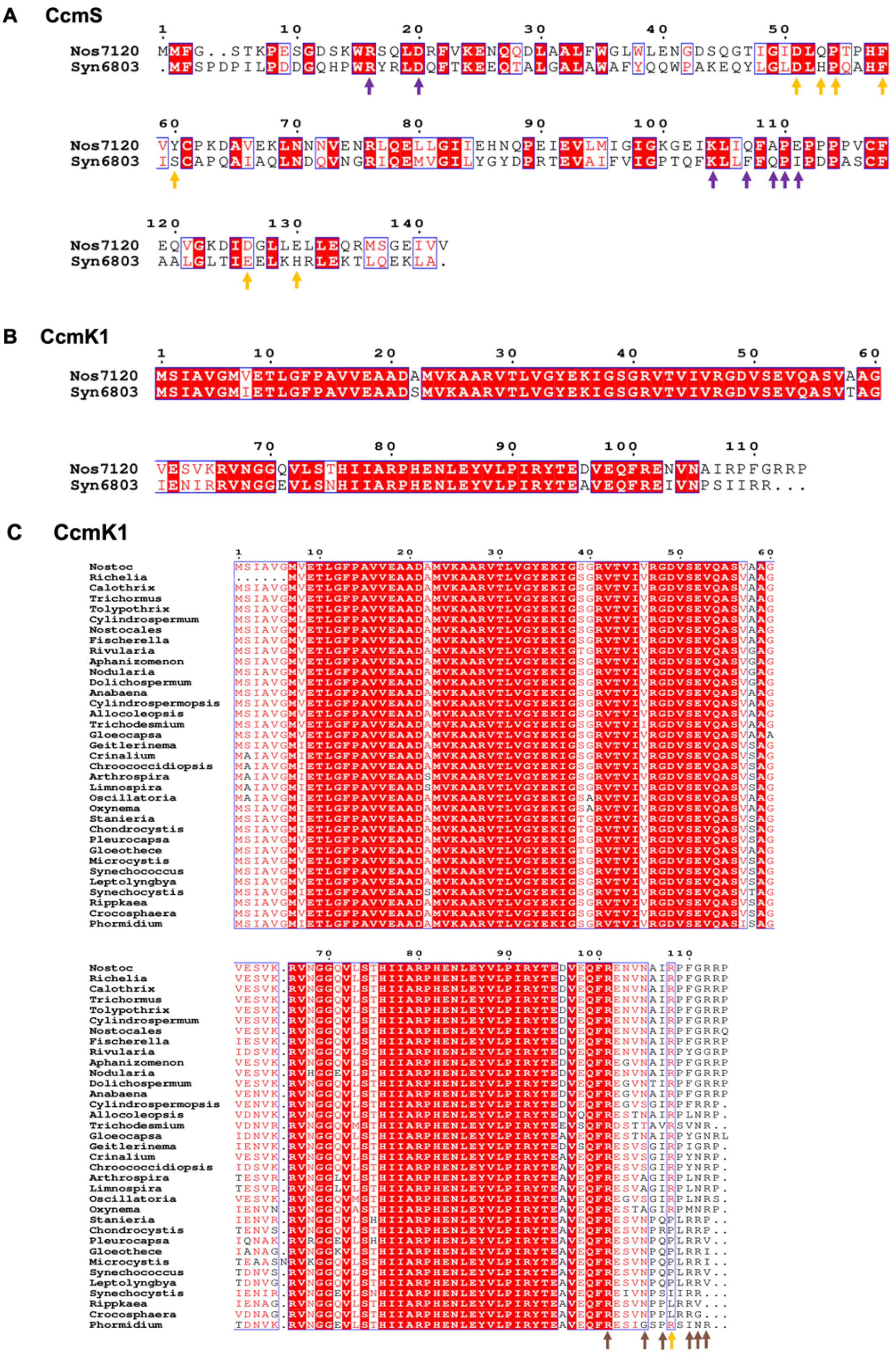

Supplementary Figure S6. Sequence alignment analysis of CcmS and CcmK1 from *Nos*7120 and *Syn*6803, as well as CcmK1 from diverse cyanobacterial species. High similar residues (similarity higher than 70%) are colored in red and framed in blue, residues with strict identity are in white on a red background. **A,** Sequence alignment analysis of CcmS in *Nos*7120 and *Syn*6803. The amino acids Arg16, Asp 20, Lys104, Gln107, Ala109, Pro 110, Glu111 involved in the interaction at the CcmS dimer interface are marked by purple arrows, and the amino acids Asp51, Gln53, Tyr60, His57, Phe58, Asp126 and Gln130 involved in the interaction of CcmS with CcmK1-C15 are marked by orange arrows. **B,** Sequence alignment analysis of CcmK1 in *Nos*7120 and *Syn*6803. **C,** Sequence alignment analysis of CcmK1 from diverse cyanobacterial species. The key amino acid Arg108 for the interaction of CcmK1-C15 with neighboring CcmS is marked by an orange arrow, and the Arg101, Ile107, Asn105, Phe110, Gly111, and Arg112 residues of CcmK1 involved in the interaction of CcmS with CcmK1-C15 are marked by brown arrows.

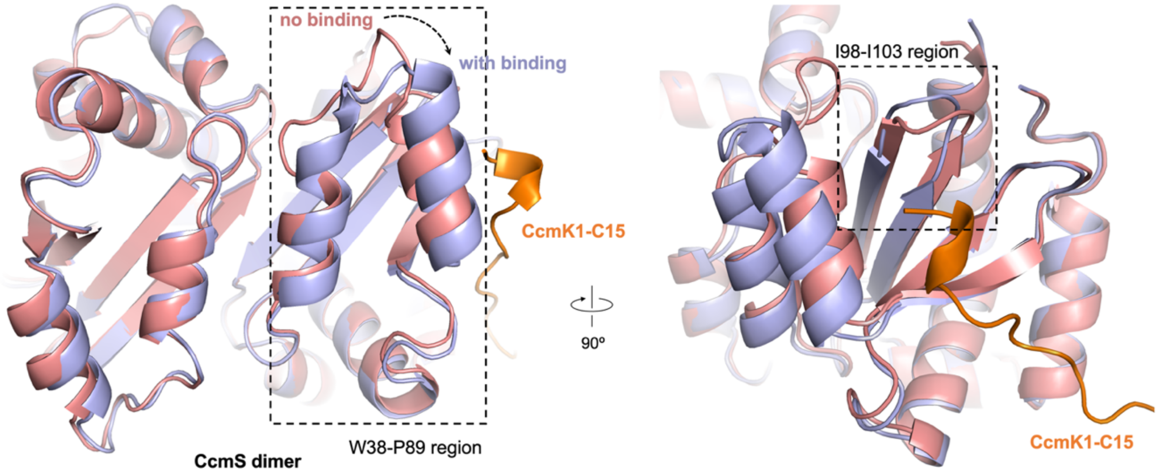

Supplementary Figure S7. Comparison of the crystal structures of CcmS and the CcmS/CcmK1-C15 complex indicates the conformational changes of the CcmS dimer resulted from the binding of CcmK1-C15. These conformational changes occur predominantly in the W38-89 (left) and I98-I103 (right) regions. The structures of CcmS and CcmS/CcmK1-C15 complex are respectively shown in red and purple ribbon representation, respectively. CcmK1-C15 is colored in orange.

Supplementary Table S1. Crystal parameters, data collection and structure refinement.

Parameters	CcmS (PDB ID: 8ZLH)	CcmS/CcmK1-C15 complex (PDB ID: 8ZLZ)
**Diffraction data**		
Space group	$P2_12_12_1$	$P4_12_12$
Unit cell		
a, b, c (Å)	40.54, 74.63, 93.57	67.49, 67.49, 118.11
α, β, γ (°)	90.00, 90.00, 90.00	90.00, 90.00, 90.00
Resolution range (Å)	35.63-1.99 (2.09-1.99)[a]	58.61-1.67 (1.76-1.67)
Multiplicity	12.6 (11.9)	22.0 (8.3)
Completeness (%)	100.0 (100.0)	98.5 (90.6)
R-merge[b]	0.089 (0.93)	0.091 (0.63)
I/sigma(I)	18.7 (2.7)	27.8 (2.8)
Total Reflections	256710 (34294)	709345 (35037)
Unique reflections	20318 (2894)	32268 (4199)
**Refinement statistics**		
R-work	0.20	0.19
R-free	0.24	0.22
RMSD from ideal geometry		
Bond lengths (Å)	0.008	0.01
Bond angles (°)	1.08	1.12
Ramachandran plot (%)		
Favored	97.66	98.14
Allowed	2.34	1.86
Outliers	0.00	0.00
Overall B-factors ($Å^2$)	42.32	17.77

[a]The values in parentheses refer to statistics in the highest bin.

[b]$R_{merge}=\sum_{hkl}\sum_i|I_i(hkl)- <I(hkl)>| / \sum_{hkl}\sum_i I_i(hkl)$, where $I_i(hkl)$ is the intensity of an observation and $<I(hkl)>$ is the mean value for its unique reflection; Summations are over all reflections.

Supplementary Table S2. Primers used in this study.

Primers	Sequence (5'-3')
**Gene cloning primes**	
ccmM-N-F	CTTTAAGAAGGAGATATACATATGTGGAGCCACCCGCAGTTTG AAAAAGCGG
ccmM-N-R	GTGGTGGTGGTGCTCGAGTTATACTTCGGAGCTACGTTCTAA
**Primers for gene validation**	
T7	TAATACGACTCACTATAGGG
T7-ter	TGCTAGTTATTGCTCAGCGG
